# Patterns of and Motivations for Concurrent Use of Video Games and Substances

**DOI:** 10.3390/ijerph8103999

**Published:** 2011-10-19

**Authors:** Geoffrey L. Ream, Luther C. Elliott, Eloise Dunlap

**Affiliations:** 1 School of Social Work, Adelphi University, 1 South Avenue, Garden City, NY 11530, USA; 2 Institute for Special Populations Research, National Development and Research Institutes, 8th Floor, 71 West 23rd Street, New York, NY 10010, USA; E-Mails: luther@nyu.edu (L.C.E.); dunlap@ndri.org (E.D.)

**Keywords:** video games, caffeine, tobacco, alcohol, marijuana, addiction, dependence

## Abstract

“Behavioral addictions” share biological mechanisms with substance dependence, and “drug interactions” have been observed between certain substances and self-reinforcing behaviors. This study examines correlates of patterns of and motivations for playing video games while using or feeling the effects of a substance (concurrent use). Data were drawn from a nationally-representative survey of adult Americans who “regularly” or “occasionally” played video games and had played for at least one hour in the past seven days (n = 3,380). Only recent concurrent users’ data were included in analyses (n = 1,196). Independent variables included demographics, substance use frequency and problems, game genre of concurrent use (identified by looking titles up in an industry database), and general game playing variables including problem video game play (PVP), consumer involvement, enjoyment, duration, and frequency of play. Exploratory factor analysis identified the following dimensions underlying patterns of and motivations for concurrent use: pass time or regulate negative emotion, enhance an already enjoyable or positive experience, and use of video games and substances to remediate each other’s undesirable effects. Multivariate regression analyses indicated PVP and hours/day of video game play were associated with most patterns/motivations, as were caffeine, tobacco, alcohol, marijuana, and painkiller use problems. This suggests that concurrent use with some regular situational pattern or effect-seeking motivation is part of the addictive process underlying both PVP and substance dependence. Various demographic, game playing, game genre of concurrent use, and substance use variables were associated with specific motivations/patterns, indicating that all are important in understanding concurrent use.

## 1. Introduction

Although the potential of substance use to add to the experience of self-reinforcing behaviors is recognizable by anyone who smokes tobacco while playing cards or drinks wine at the theater, there is little systematic research on it. Research should address this issue because, at least in the case of behaviors with addictive potential, concurrent substance use might not be entirely benign. A recent review found problem gambling to share nosological, clinical, and biological features with substance use disorders [1], and laboratory studies find alcohol exacerbates problem gambling [2–4]. Several studies, in fact, have found “behavioral addictions” [5] and substance use problems to share common biological pathways, including the endogenous cannabinoid [6], dopamine [7], and hypocretin [8] systems underlying reward and arousal.

The present study is concerned with video game play, which has demonstrated effects on the brain over time similar to substance addiction [9–11]. Video game “addiction” is not universally accepted [12–14], and a lack of consensus on how to measure it makes estimating prevalence difficult [15,16]. However, research has generally affirmed the existence of clinically significant problem video game play, with prevalence estimates of problem gaming behaviors hovering between 4.9% and about 9% among video gamers internationally [15,17–21]. Smaller numbers have been found to fit stricter criteria for dependence, e.g., 2–5% of children and youth overall in a recent review [15], and 3% of male and 0.3% of female adolescents in a German national study [22]. Problem video gaming behavior is a growing source of scholarly and clinical concern [23,24], with an American Medical Association report calling for more research on it [25]. Findings from treatment of problem video gaming behavior further underscore its biological dimensions [15]: Video games are associated with development of attention problems in children [26,27], although this relationship is complex and involves other factors [28], and dopaminergic medications indicated for ADHD and substance dependence have been shown to remediate problem video gaming behavior [29,30].

Problem video gaming behavior is also associated with measures of “addiction” to various substances, even caffeine [18,31–33]. This leads to the concern that playing video games while using or feeling the effects of substances—called “concurrent use” in this study—may create a complementary effect similar to the one found with gambling [2–4]. To the extent that players experience this “drug interaction” intentionally through concurrent use, it is logical to ask about patterns of and motivations for this behavior [24]. These considerations are potentially important to addiction specificity [34], *i.e.*, differential development of specific patterns of addictive behaviors based on attraction to the behavior, shared experiences with other participants in the behavior, expectations for outcomes of the behavior, and other factors. For example, concurrent use with any conscious, regular situational pattern or effect-seeking motivation is probably associated with greater degrees of problem use of both video games and substances than merely coincidental concurrent use. Also, specific substances of concurrent use, demographic groups of concurrent users, or social situations of concurrent use may be associated with some concurrent use patterns/motivations but not others. Video game genres [35–38] involved in concurrent use may be differentially compatible with specific patterns/motivations of concurrent use.

This study, accordingly, explored patterns/motivations of concurrent use in the context of general patterns of game playing and substance use behavior. In addition to simple frequency and duration of concurrent use, we considered preferred context of concurrent use (alone or with certain friends) and several effect-seeking motivations, including self-medication of loneliness or depression, using substances to enhance game experience, using substances to cope with game-related frustration, using video games to pass time while feeling effects of substances, and using video games to cope with substance withdrawal. Our analyses first distinguished common factors underlying patterns of and motivations for concurrent use. Then, we explored potential correlates of both frequency variables and these factors among demographics, general game playing behavior, genre of concurrent use, and substance of concurrent use, including substance use problems.

## 2. Methods

### 2.1. Participants and Recruitment

Participants were a subset of a nationally representative KnowledgePanel^®^ maintained by the commercial online research survey provider Knowledge Networks (KN). KN selects panel members via random-digit dial and address-based sampling, provides computers and internet access if needed, establishes informed consent, and collects demographics. KN randomly recruits panel members via e-mail for client surveys (e.g., the present study) which they incentivize with “points” toward cash and other rewards. KN offers the option of screening the randomly selected panel members and only allowing them to participate if they meet client-specified criteria. For this survey, 15,642 e-mails were sent to panel members ages 18 and over, and 9,215 (59%) completed the screening instrument. The screener asked whether participants “regularly,” “occasionally,” or “never” participated in 11 different hobby activities in the past year, including video games. Participants who responded “regularly” or “occasionally” about video games were then asked how many hours they played in the past 7 days. Participants who reported one or more hours (n = 3,380; 37%), were allowed to take the survey. The screening and survey were conducted in either English or Spanish. Median completion time was 10 minutes, the maximum feasible given budgetary and methodological constraints. The protocol for this study was reviewed and approved by all investigators’ Institutional Review Boards.

### 2.2. Measures

#### Demographics

Age, race/ethnicity, gender, education, income, metropolitan statistical area (MSA) resident status, and employment status were taken from Knowledge Networks’ basic demographic survey.

#### Video game days used, hours/day used, and enjoyment

Participants were asked to list up to five video game titles they had “spent a lot of time playing in the past 12 months.” For each title, they were asked how many days of the past 30 they had played it, how many hours they played on days they played it, and how much they enjoyed it. Enjoyment was a single 7-point Likert scale in which 1 = “it was the worst game I’ve ever played,” 4 = “about the same as most games,” and 7 = “it was my single all-time favorite.” These variables were averaged within each participant to reflect the average game that person played.

#### Consumer involvement

Participants were asked about dimensions of enthusiasm for video games with no necessary addictive connotation – attraction, centrality/importance, and self-expression [39–41] using a 3-item Likert-scale measure adapted from leisure and marketing studies, Cronbach’s α = 0.72.

#### Problem video game playing (PVP)

A 5-item version of the original 9-item Likert scale [42] was used to measure increased time spent playing (tolerance), difficulty controlling time spent playing, restlessness/irritability when can’t play (withdrawal), play to relieve negative emotions (self-medication), and disregarding negative consequences of play, Cronbach’s α = 0.76.

#### Video game genres of concurrent use

Of 7,203 titles from 3,380 participants, 6,056 from 2,885 participants could be clearly distinguished as valid titles of single games or game series with identical genre descriptors (e.g., professional football simulations updated annually to include current years’ players). Valid titles were those that could be found in GameFaqs [43], an exhaustive database of user-generated content maintained and edited by an industry group. Invalid entries included overly-broad categories of games, names of game platforms, or qualitative responses. Participants listed a total of 1,335 different valid titles, ranging in frequency from 1 to 340. Each title’s genre was coded as the major category under which it was listed in GameFaqs, with some categories broken into theoretically significant subcategories, as follows: Action-adventure, massively multiplayer online role-playing games (MMORPG’s), other role-playing games (RPG’s), first-person shooter (FPS), other shooter, gambling, real-time Strategy (RTS), other strategy, board/card games, sports general, other sports, puzzle, rhythm, driving, platformer, and a catch-all category of other genres including titles that were valid but belonged to genre categories with 10 or fewer titles or 30 or fewer players (e.g., fighting).

Participants were also asked whether they had played each game that they listed while using or feeling the effects of substances. For each of 16 dummy variables for genre of concurrent use, participants were coded 1 if they reported playing a game from that genre and concurrent use with it, and 0 of they either reported playing a game from that genre with no concurrent use or did not report playing that genre.

#### Substance use frequency and use problems

Measures were adapted from the National Survey of Drug Use and Health (NSDUH, [44]). Participants chose substances they had used in the past 30 days from a list. For each substance used in the past 30 days, participants were asked on how many of the past 30 days they had used and presented with a series of abuse/dependence symptoms. In order to keep the survey within the median length of 10 minutes, measures were shortened by selecting those with the highest correlation to an underlying dimension of abuse/dependence from factor analysis of data from a related study (a computer assisted personal interview survey supported by the same grant as the present study; data analyses are underway) which used the full measures. Measures for this study included five dichotomous items for caffeine (tolerance, difficulty controlling use, desire to quit/cut down, withdrawal, disregarding negative emotional/physical health consequences), four Likert-scale items for tobacco (withdrawal, craving, worry over running out, tolerance), and seven dichotomous items each for alcohol, marijuana, painkillers, and sedatives (tolerance, difficulty controlling use, desire to quit/cut down, withdrawal, disregarding negative emotional/physical health consequences, neglecting positive activities, and spending a lot of time obtaining or using). Reliability was found to be adequate according to comparative fit indices from confirmatory factor analyses for each substance: caffeine: 0.962, tobacco: 0.990, alcohol: 0.997, marijuana: 0.998, painkillers: 0.979, sedatives: 0.994.

#### Concurrent use patterns and motivations

These items assessed endorsement of various patterns of and motivations for concurrent use of video games and substances identified in previous research and pilot qualitative data. Participants who reported recent concurrent use were asked how many days of the past 30 and the average number of hours on each of those days they spent concurrently using, and to respond to each of the following on a 5-point Likert scale from 1 = “Not at all true” to 5 = “Extremely true”: “When you’re alone, you like to play video games and use substances,” “When you get together with certain friends, you often use substances and play video games,” “You use video games and substances together to help cope with loneliness or depression,” “Certain substances really enhance your experience of certain video games,” “When video games become frustrating, you use substances to calm down,” “You play video games to pass the time while feeling the effects of a substance,” “You play video games to get through withdrawal, being hung over, or coming down from a substance”.

### 2.3. Approach to Analyses

Income was categorized into increments that were increasingly larger further up the scale until “$175,000 or more.” Employment was collapsed into categories of (1) working, either for wages or self-employed, or (2) non-working for any reason, e.g., disability, retirement, layoff, *etc*. Because these variables were from KN’s demographic database, no data are missing on them.

Table 1 reports the most parsimoniously interpretable set of results from several exploratory factor analyses conducted in STATA 12 of the Likert-scale pattern/motivation variables. Similar structure emerged from procedures involving all combinations of Kaiser normalization on or off, maximum likelihood or principal factors estimation, and promax or (oblique) Bentler’s invariant pattern simplicity rotation. Most other estimation and rotation methods produced either a single factor or so many cross-loaded items that simple structure was not achieved. Once the factor structure was settled upon, confirmatory factor analysis was run in MPlus 6.0 using maximum likelihood estimation with robust standard errors.

The analysis presented in Table 2 is a single multivariate model estimated in MPlus 6.0 with the frequency indicators and motivation factors as dependent variables and all demographic, game playing behavior, game genre, and substance use/problem variables as independent variables. We specified the model so that dependent variables were allowed to correlate. In order to include all cases in this single omnibus analysis, zeroes were imputed for substance use variables for non-users of the substance in question. Like the factor analysis reported in Table 1, the analysis reported in Table 2 is also the most parsimoniously interpretable of several alternative specifications, which variously involved latent indicators for some or all of the composite variables. All alternatively specified models had adequate fit [45] and similar patterns of significant coefficients for independent variables, indicating that our results are robust.

All analyses employed post-stratification weights provided by Knowledge Networks so that estimates more accurately reflect what would have been obtained from a true random sample of English- and Spanish-speaking American adult video game players [46]. Only weighted point estimates and hypothesis tests are presented in the results section.

## 3. Results

Cases were valid for analysis if they reported at least one game title for which a genre could be discerned and recent use of at least one game with a substance (or *vice versa*), resulting in a total sample size of 1,196 concurrent users. Their mean age was 40.6 (SD = 15.1) and mean educational level (operationalized as an ordinal variable) corresponded to “some college, no degree,” and mean income level (also ordinal) corresponded to $35,000–$39,999. The majority of concurrent users were white (76%), male (64%), living in an MSA (82%) and engaged in work for regular wages (53%). The average participant reported on 2.5 games out of the 5 the survey allowed, played their average game 11.5 days out of the past 30 with 2.9 hours per day played. They reported enjoying their average game “more than most games”.

Mean consumer involvement was 2.3 (SD = 0.9), and mean problem video game play was 1.7 (SD = 0.7). Caffeine use in the past 30 days was reported by 78% (n = 940), tobacco by 45% (n = 540), alcohol by 43% (n = 512), marijuana by 11% (n = 136), painkillers by 11% (n = 131), and sedatives by 4% (n = 51). The modal response to all substance dependence measures was 1 on a scale of 1–5 for tobacco and 0 symptoms for all other substances. Means for Likert-scaled concurrent use situations and complementary use motivations variables were close to 1 on a scale of 1–5, and 34% of participants responded “not at all true” to all of them, indicating that about two-thirds of concurrent users had any conscious situational pattern or effect-seeking motivation for their concurrent use.

Table 1 reports the results of an exploratory factor analysis (EFA) of the pattern/motivation variables. Use while alone, use to cope with loneliness/depression, and use to pass time while feeling the effects of a substance loaded > 0.5 on a factor of concurrent use to pass time or regulate negative emotion. Use with certain friends and belief that certain substances enhance the experience of certain games loaded > 0.5 on a factor of concurrent use to enhance an already enjoyable or positive experience. Use of substances to cope with game-related frustration and use of video games to cope with hangover or other substance withdrawal loaded > 0.5 on a factor of concurrent use to remediate each other’s undesirable effects (*i.e.*, video games to remediate the undesirable effects of substances and *vice-versa*). A confirmatory factor analysis of this 3-factor solution (using maximum likelihood estimation with robust standard errors) produced CFI = 0.962, standardized factor loadings between 0.624 and 0.764, and correlations among factors between 0.643 and 0.771. Factor scores from the EFA were saved and used as dependent variables in the following analysis.

Table 2 reports the results of a single multivariate analysis with days of concurrent use, hours/day of concurrent use, and the three pattern/motivation factors as dependent variables and demographics, general game playing variables, genres of concurrent use, and substance use frequency and problem use indicators as independent variables. Among demographic factors, younger age was associated only with concurrent use to enhance an already enjoyable or positive experience, lower education was associated only with longer concurrent use sessions, and non-working status was associated only with more days of concurrent use and use to pass time or regulate negative emotion.

Among game playing variables, days of video game play was only associated with days of concurrent use. Consumer involvement and game enjoyment did not have significant direct effects on any pattern/motivation. Hours/day of video game play and PVP were, however, associated with hours/day of concurrent use and all three pattern/motivation factors. Given that all five general game playing variables were correlated, with weighted Pearson r’s between 0.13 and 0.59, all p’s < 0.0001, it is suggestive that hours/day of video game play and PVP stood out as significant after statistical control. It is also noteworthy that, after controlling for all of these game playing factors, certain genres of concurrent use emerged as particularly compatible with certain patterns/motivations. The only genres not associated with either frequency variable or any pattern/motivation factor were MMORPG’s, gambling, RTS, sports-general. The only dependent variable with no genre uniquely associated with it was concurrent use of video games and substances to remediate each other’s undesirable effects.

With respect to substance use, almost all associations specified between indicators of substance use problems and pattern/motivation factors were significant. Negative coefficients for substance use frequency variables should be interpreted in light of bivariate analyses (not shown) in which substance use frequency was generally unrelated to concurrent use patterns/motivations; their significance in the multivariate context may be because of these indicators’ collinearity with the use problems variables. The only substance of concurrent use not uniquely associated with any concurrent use frequency variable or pattern/motivation factor was sedatives.

## 4. Discussion

This study found that concurrent use with some situational pattern or effect-seeking motivation behind it is, like PVP [21], not universal among those who engage in the prerequisite behavior, but still observable in an appreciable fraction: 42% of the entire valid sample of video gamers were concurrent users, and 66% of concurrent users at least partially endorsed one or more patterns/motivations. The reliable correlation of most patterns/motivations with PVP and substance use problems suggests that concurrent use is part of a shared underlying addictive process [47], as other research on concurrent use of substances with self-reinforcing behaviors [3–6,48,49] suggests.

Moreover, demographics, genre of concurrent use, and substance of concurrent use variables were all uniquely and differentially related to concurrent use frequency and patterns/motivations. Concurrent use to enhance an already enjoyable or positive experience – either being with certain friends, or use of certain substances to enhance the experience of certain games – was more strongly endorsed by younger participants. An example of this factor’s operation in context might be evident in ethnographic findings on parties among young marijuana users, where video games were commonly part of the entertainment [50]. Others of our results indicate differences among concurrent users in how concurrent use fits into the context of their lives, including that respondents with lower degrees of education reported longer hours/day of concurrent use, and non-working respondents reported greater degrees of use to pass time or regulate negative emotion.

Both intuitive and counterintuitive findings emerged for genre of concurrent use, which may further inquiry into connections between specific game features and problem video gaming behaviors [35]. In spite of the problem use potential of MMORPG’s [37,38], concurrent users of MMORPG’s were no more likely than others to endorse particular concurrent use patterns or motives. Other (non-MMO) RPG’s, in contrast, were associated with more days of concurrent use, greater endorsement of use to pass time or regulate negative emotion, and higher degrees of concurrent use to enhance an already-positive or enjoyable experience. This may have to do with differences in game play—one possible explanation is that it is easier to use a substance with non-MMO RPG’s because players can take non-MMO RPG’s at their own pace and do not have to remain engaged with other players in real time. First-person shooters (FPS) were also distinct from other shooters, significantly related to the same factors as RPG’s while other shooters were not. This may be because many FPS games are designed for team or competitive play among players gathered in person, and concurrent users use substances at these gatherings. Alternatively, perhaps the intensity of seeing the game from the character’s perspective—e.g., bullets flying right at the player’s face—occasionally causes a concurrent user to want something to calm them down. Board/card games were also associated with both concurrent use to pass time or regulate negative emotion and concurrent use to enhance an already-enjoyable or positive experience while gambling games were not, perhaps because even fictional money is enough to alter the experience of a board/card game. Finally, the category of other sports games, which included mostly multiplayer team sports simulations, was associated with concurrent use to enhance a positive experience, while the mostly motion-control sports-general games were not. This could be because of a special association between team sports simulation games and gatherings involving substance use, or perhaps because use of some substances makes the coordinated full-body motion required to play motion control games more difficult.

Substance use problems were not positively related to simple frequency or duration of concurrent use. They were, however, reliably related to the pattern/motivation factors, with some exceptions: For example, caffeine is understandably not helpful for calming down from video game related frustration (“rage quit” in gamers’ own parlance), nor does its withdrawal syndrome usually cause enough impairment to distract from normal activities. These and other non-significant findings among the general pattern of significant associations between substance use problems and pattern/motivation factors are potentially relevant to the concern of addiction specificity [34], in that every behavior/substance combination may have its own unique complementarity of effects, context of use, expectations among users as to what they will get out of it, and other unique considerations which ultimately affect which patterns of addictive behaviors will develop. Our measures were not, however, set up to directly operationalize addiction specificity itself, e.g., clinically significant substance use problems without PVP and *vice-versa*. It will be up to future work to explore those associations. Ideally, questions about media and other behavioral addictions would be added to existing nationally representative panel studies addressing substance use and health like NSDUH [44] or Monitoring the Future [51].

One way in which this study uniquely contributes to the literature is that, rather than focusing on specific game genres, game features, playing behaviors, or substances, it allowed for an open field of possible correlates. It also departed from this area’s frequent focus on youth problem behavior [15] by including a nationally representative sample of adults. It is limited, however, in that our survey’s 10-minute median length meant that established measures [42,44] had to be abridged. Hence, our references to “substance use problems” rather than actual abuse/dependence diagnoses. Also, as an internet survey, it was vulnerable to same risk of invalid response endemic to any survey method that does not have interviewers engaged in-person with participants. One symptom of this was that 15% of our respondents did not provide even one valid video game title. The online nature of the survey in and of itself, however, probably cannot be argued to be a limitation, as experiments have shown point estimates to be consistent across methods of obtaining random, representative survey samples [52]. Finally, our cross-sectional data can only contemporaneous association; they cannot address development of motivations for and patterns of behavior over time.

Although this study’s results did not coalesce into a simple story, we believe that this is actually a more authentic representation of the phenomenon under study than could have been achieved by focusing on specific demographics, substances, gaming behaviors, and game genres, or by oversimplifying the question into “are video games addictive?” Our results represent a complex issue in its complexity, suggesting that social situation, playing behaviors, genre and substance of concurrent use, and motivations for concurrent are all potentially relevant to the effect of concurrent use on individuals’ lives.

## Figures and Tables

**Table 1:** Exploratory factor analysis derivation of factor score indicators for patterns of and motivations for concurrent use.

		Pass time or regulate negative emotion	Enhance positive experience	Remediate each other’s a undesirable effects	Uniqueness
Factor loadings:	While alone	**0.78**	0.12	−0.23	0.45
	Cope with loneliness/depression	**0.70**	−0.26	0.26	0.43
	Pass time while feeling effects of substance	**0.51**	0.20	0.09	0.49
	With certain friends	−0.01	**0.72**	0.09	0.44
	Substances enhance experience of games	0.09	**0.55**	0.18	0.51
	Substance to cope with game-related frustration	0.04	0.04	**0.60**	0.58
	Play games to cope with substance withdrawal	−0.08	0.13	**0.63**	0.59

Correlation matrix:	Pass time or regulate negative emotion	1	0.63	0.63	
	Enhance positive experience	0.63	1	0.38	
	Remediate each other’s undesirable effects a	0.63	0.38	1	

	Proportion of variance explained	0.68	0.53	0.52	

Exploratory factor analysis using maximum likelihood estimation produced three factors with positive Eigenvalues; results shown are after oblique Bentler’s invariant pattern simplicity rotation with Kaiser normalization applied to 3-factor solution.

a Refers to use of video games to remediate the undesirable effects of substances, and vice versa.

**Table 2:** Standardized coefficients from multivariate model predicting days of concurrent use, hours/day of concurrent use, and pattern/motivation factors from demographics, game playing variables, and genres/substances of concurrent use.

		Days of concurrent use	Hours/day concurrent use	Pass time or regulate negative emotion	Enhance positive experience	Remediate each other’s undesirable effects
Age		0.04	0.07	0.03	−0.09 **	−0.03
Education		0.04	−0.11 **	−0.01	−0.04	0.02
Race: Black		−0.06 *	0.15 *	0.06 +	0.00	0.04
Race: Asian		−0.08*	−0.04	−0.01	0.05	0.04
Race: Native		−0.04	0.03	−0.04	−0.06 *	−0.02
Race: Other		−0.05 **	−0.01	0.00	−0.01	−0.01
Non-working		0.07 *	0.00	0.07 *	0.04	0.04
Days played average game		0.34 ***	0.06	0.03	−0.01	0.00
Hours/day played avg. game		0.01	0.23 ***	0.17 **	0.17 ***	0.17 *
Problem video game play		0.08 +	0.12 *	0.24 ***	0.13 **	0.26 ***
Genre: Action-adventure		0.06 *	0.08 *	0.04	0.08 *	0.01
Genre: Other RPG (not MMO)		0.08 **	0.03	0.08 *	0.10 **	0.00
Genre: First-person shooter (FPS)		0.11 ***	−0.01	0.10 **	0.10 *	0.06
Genre: Other shooter		0.06 *	0.07	−0.01	−0.01	−0.03
Genre: Other strategy (not RTS)		0.10 ***	0.01	−0.01	0.02	−0.04
Genre: Board/card		0.15 ***	0.03	0.10 ***	0.07 **	0.02
Genre: Other sports		0.07 **	0.00	0.04	0.11 **	0.01
Genre: Puzzle		0.15 ***	0.00	0.00	−0.01	−0.04
Genre: Rhythm		0.06 *	0.03	0.04	0.03	0.02
Genre: Platformer		0.05 *	0.02	0.08 *	0.02	0.03
Genre: Other		0.10 ***	0.03	0.03	−0.02	0.03
Caffeine days used		0.17 ***	0.08 +	−0.08 *	−0.07 *	−0.09 *
Caffeine use problems		−0.03	−0.08 +	0.12 ***	0.08 *	0.05
Tobacco use problems		0.05	0.16 +	0.19 **	0.05	0.23 **
Alcohol days used		0.07 *	−0.04	0.05	0.08 **	−0.02
Alcohol use problems		−0.11 ***	0.00	0.08 *	0.09 *	0.05
Marijuana days used		0.16 ***	−0.02	0.07	0.10 +	−0.06
Marijuana use problems		−0.08 *	0.06	0.15 *	0.21 **	0.15 *
Painkiller days used		−0.01	−0.08 *	−0.14 ***	−0.08 **	−0.11 ***
Painkiller use problems		0.01	0.00	0.24 ***	0.12 *	0.34 ***
				
Correlations among dependent variables:	Days concurrent use	1	0.10 **	0.16 ***	0.03	0.03
	Hours/day concurrent use	0.10 **	1	0.06	0.04	0.01
	Pass time/regulate emotion	0.16 ***	0.06	1	0.68 ***	0.70 ***
	Enhance pos. experience	0.03	0.04	0.68 ***	1	0.46 ***
	Remediate undesirable eff.	0.03	0.01	0.70 ***	0.46 ***	1
				
Variance explained (R^2^)		0.38 ***	0.22 ***	0.45 ***	0.42 ***	0.38 ***

Overall model fit:		CFI > 0.999, RMSEA < 0.001, SRMR < 0.001				

Coefficients omitted from presentation in the table (they are included in the analysis) because p > 0.05 for all dependent variables include income; gender; race: Latino; MSA non-residence; enjoyment of average game; consumer involvement; the following genres: MMORPG, Gambling, RTS, Sports-general, Driving; days of tobacco use; days of sedative use; and sedative use problems. Reference category for race is white.

+ p < 0.10,

* p < 0.05,

** p < 0.01,

*** p < 0.001.
